# Effect of antibiotics and NSAIDs on cyclooxygenase-2 in the enamel mineralization

**DOI:** 10.1038/s41598-018-22607-z

**Published:** 2018-03-07

**Authors:** Clara Serna Muñoz, Amparo Pérez Silva, Francisco Solano, María Teresa Castells, Ascensión Vicente, Antonio José Ortiz Ruiz

**Affiliations:** 1 Department of Integral Paediatric Dentistry, Faculty of Medicine, University of Murcia, Murcia, Spain; 2 Department of Biochemistry, Molecular Biology & Immunology, Faculty of Medicine, University of Murcia, Murcia, Spain; 3 Department of Cell Biology and Histology, Faculty of Medicine & Research Supporting Facility, University of Murcia, Murcia, Spain; 4 Department of Orthodontics, Faculty of Medicine, University of Murcia, Murcia, Spain

## Abstract

The objective of this study was to determine whether the use of the most commonly prescribed antibiotics and non-steroidal anti-inflammatory drugs in childhood could disturb enamel mineralization. Forty-two Swiss mice were divided into seven groups: controls; amoxicillin; amoxicillin/clavulanate; erythromycin; acetaminophen; ibuprofen and celecoxib, to inhibit cyclooxygenase 2 (COX2). SEM-EDX analysis was conducted on all cusps of the third molars. Calcium (Ca), phosphorus (P), aluminum, potassium, sodium, magnesium and chlorine were quantified. The stoichiometric Ca/P molar ratios were calculated. Immunohistochemical quantification of COX2 in incisors was carried out by image analysis using COX2-specific immunostaining. Groups treated with antibiotics showed no significant differences in the content of the chemical elements. Only acetaminophen and celecoxib showed a significant decrease in Ca and P compared with the control samples. Ca/P ratios showed no difference. Groups treated with amoxicillin, amoxicillin/clavulanate, erythromycin and acetaminophen showed significantly lower amounts of immunoreactive COX2 at the enamel organ maturation stage of the mouse incisors. Our results suggest that COX2 is involved in the maturation stage of the enamel organ and that its inhibition would appear to alter amelogenesis, producing hypomineralization.

## Introduction

Enamel defects are classified as qualitative (enamel hypomineralization) or quantitative (enamel hypoplasia). Hypoplasia is a reduction in enamel thickness, while hypomineralization is characterized by normal enamel thickness but defective quality^1^. The etiology of both dental enamel defects may be hereditary, systemic, local or idiopathic^2^.

Molar incisor hypomineralization (MIH) is a qualitative idiopathic enamel defect of one to four first permanent molars and is frequently associated with the incisors. It may be diagnosed as soon as the first molars have erupted^3^.

MIH is characterized by defective enamel quality and is thought to be caused by disruption of the final two stages of amelogenesis: the transition and maturation stages^4^. Amelogenesis is divided into three main stages: secretory, transition, and maturation. During the secretory stage, ameloblasts secrete an extracellular protein matrix (mostly consisting of amelogenins with smaller amounts of other proteins) and matrix metallopeptidase 20 (MMP20) for the restricted digestion and assembly of the structural matrix. Concomitantly with proteolysis, mineralized material is deposited for crystal growth. During the transition stage, ameloblasts change their morphology and about 25% undergo apoptosis. During maturation, the enamel’s protein content further decreases due to the action of other proteases secreted at this stage [mainly kallikrein related-peptidase 4 (KLK4)], and more inorganic apatite material is deposited on the preexisting preformed enamel crystals to achieve the characteristic thickness and width^5,6^.

MIH is highly prevalent worldwide. Schwendicke *et al*.^7^ found a prevalence of 13.1% (11.8–14.5%), with significant differences between superregions, regions and countries, with low- and middle-income countries shouldering the majority of the burden.

The etiology of MIH is unknown, but it has been associated with various prenatal, perinatal, and postnatal problems. Postnatally, MIH may be associated with childhood diseases^8^ and with medication administered during the first three years of life, when molar and incisor calcification occurs^9,10^.

A recent extensive systematic literature review of the etiology of MIH in relation to medication intake by Serna *et al*.^11^ included more than 1,000 papers published between 1965 and 2014, in order to establish a preventive protocol for patients at potential risk of MIH. Differing methodologies and terminology hindered the extraction of clear results and pointed to the need for well-designed studies using animal models to advance understanding of the relationships between MIH and medication.

In this context, the main objective of this paper was to determine whether the use of the most commonly prescribed antibiotics in childhood, including amoxicillin alone or supplemented with potassium clavulanate, erythromycin^12^, and the frequently-administered non-steroidal anti-inflammatory drugs (NSAIDs) acetaminophen (paracetamol) and ibuprofen^13^, disturb molar mineralization in young mice.

NSAIDs exert their anti-inflammatory effect by inhibiting COX and prostaglandin synthesis, but there are at least two different enzymes involved in this activity; constitutive COX1 and inducible COX2, which is more related to inflammatory processes. COX2 is also an essential mediator of bone formation and bone resorption^14^. Therefore, to explore the cellular effects of the two NSAIDs, celecoxib, a selective inhibitor of COX2, was also studied^15,16^.

## Materials and Methods

### Animals and Surgical Procedure

Animals were bred and fed *ad libitum* in the animal facility of the University of Murcia (Murcia, Spain). The animals were treated according to Spanish and European Community guidelines for the bioethical use of animals for scientific experimentation (RD 53/2013, Law 32/2007, and European Directive 2010/63/EU). All experiments were performed in accordance with relevant guidelines and regulations. The study was approved by the University of Murcia bioethics committee (Ref. 675/2016).

Forty-two Swiss male, recently-weaned mice (21 days old, weight 15–20 g) were randomly divided into seven groups of six: (a) control group, without medication; (b) amoxicillin group, treated with 5 mg/day of amoxicillin; (c) amoxicillin/clavulanate group, treated with 2.5/0.31 mg/day; (d) erythromycin group, treated with 5 mg/day; (e) acetaminophen group, treated with 5 mg/day; (f) ibuprofen group, treated with 2.5 mg/day; (g) celecoxib group, treated with 0.12 mg/day. This last group was constituted in order to inhibit COX2. The doses administered were chosen as the equivalent to the normal daily doses given to children normalized according to body weight, with the exception of celecoxib, which was administered at doses extrapolated from adult doses, as this drug is not recommended in children.

All treatments continued for 30 days (until day 51 of life) and drugs were supplied daily to the animals in fresh strawberry gelatin. The same gelatin was also supplied to control mice but without medication. All animals were kept in individual cages to ensure each mouse ingested the correct dose. After 30 days, all mice were sacrificed by CO_2_ inhalation. The upper and lower jaws were removed and all soft tissue carefully cleaned by dissection. Jaw segments containing all three upper or lower molars were cut out with a rotating diamond wheel cutter under water-cooling, washed with double distilled water and left to dry at room temperature for 24 hours. Jaw segments containing incisors were immediately (<5 min postmortem) fixed in 10% buffered formalin for 15 days. The molar segments were used for energy dispersive X-ray (EDX) analysis and the incisors for immunohistochemistry analysis.

### Scanning electron microscopy–Energy dispersive X-ray analysis

When jaw segments containing molars were dried, they were affixed to scanning electron microscopy (SEM) stubs, sputter-coated with carbon and examined with a JSM-6100 JEOL SEM operating at 15 kV and 15–20 mm working distance.

Quantitative element analysis was carried out with an Oxford Instruments INCA 300 EDX System (Abingdon, Oxfordshire, UK). The element content was calculated as the relative weight percentage of the total element content (100%). The count was conducted on the buccal, lingual and central cusps of the third molars (M3) (12 measurements per mouse, 72 measurements per group). Measuring time was 100 s. The elements quantified were Ca, P, aluminum (Al), potassium (K), sodium (Na), magnesium (Mg) and chlorine (Cl). The stoichiometric Ca/P molar ratios were calculated using the following formula taking into account the respective atomic masses of the two elements: Ca/P = [Ca (% of weight)/40.08 (g/mol)]/[P (% of weight)/30.97 (g/mol)].

### Immunohistochemistry

After fixation, incisor segments were immersed in Shandon TBD-2 Decalcifier (77–80% water, 21–23% formic acid, >1% fluorad, >1% sodium citrate, >1% polyvinylpyrrolidone; Thermo Fisher Scientific, Waltham, Massachusetts, USA), which was changed daily for two weeks until decalcification. The segments were then dehydrated and embedded in paraffin. To prepare for immunohistochemical analysis, the segments were cut into microsections of 5–6 µm thickness using a RM 2155 LEICA motorized microtome (Leica Microsystems, Wetzlar, Germany): the orientation of the incisors embedded in paraffin was always the same. The microsections were placed on silane coated glass slides (StarFrost, Knittel Glässer, Braunschweig, Germany) and deparaffined (dewaxed), rehydrated, and immersed in a 3% hydrogen peroxide solution in double distilled water for 30 min to inhibit endogenous peroxidase. The microsections were then washed in in PBS, pH 7.6 and treated for antigen unmasking^17^.

For COX2-specific staining, the preparations were immersed in 0.1 M sodium citrate buffer, pH = 6 and heated for 10 min in a microwave oven at 400 W (Balay 3WM-2533, BSH, Pamplona, Spain). After cooling by several PBS washes, the preparations were blocked for unspecific binding by immersion in 0.1% normal goat serum (Sigma-Aldrich, St. Louis, MO, USA) in PBS for 1 h at room temperature. The preparations were then incubated overnight at 4 °C with 1:50 dilution of rabbit IgG against rat COX2 (Anti-COX2/Cyclooxygenase 2 antibody; ab15191; Abcam, Cambridge, UK) in PBS containing 0.1% normal goat serum. The following morning, after washing in PBS, sections were incubated in a 1:200 dilution of biotinylated goat secondary antibody against rabbit IgG (Anti-Rabbit Immunoglobulins/Biotinylated Code No. E 0432; Agilent, Santa Clara, CA, United States) in PBS containing 0.1% normal goat serum for 1 h at room temperature. COX2-specific staining was made using avidin-labeled horseradish peroxidase (Agilent, Santa Clara, CA, United States) to form the avidin-biotin complex and using 0.05% of 3,3′-diaminobenzidine and 0.03% hydrogen peroxide for 4 min as chromogen. All preparations were contrast-stained with Mayer’s hematoxylin. The primary antibody, anti-COX2, was substituted by PBS for control of the immunocytochemical technique. No staining was observed in these controls. To ready the primary antibody, mouse mammary carcinoma tissue was used: each day immune tincture was carried out, the same tissue was used as a positive control.

### Image analysis quantification of immunoreactivity

Images were captured with a Leica DFC280 digital camera mounted on a Leica DM6000B microscope. Leica Application Suite v2.5.0 software (Leica Microsystems, Wetzlar, Alemania) was used to process images. All images were captured in a single session using identical microscope illumination and camera settings. All acquisitions and protocol analyses were carried out with team members blinded to the sample group assignment. Immunoreactivity was analyzed using Leica QWin software. A digital image is a matrix of pixels in which each pixel is characterized by an allotted number ranging from 0 (black) to 255 (white), representing the intensity of transmitted light or grey level at a single point. Grey level was related to COX2-immunoreactivity. The analysis was performed using the inverted (negative) image, with the highest values corresponding to the greatest COX2 immunoreactivity. The maturation stage of the enamel organ containing COX2 reactivity was selected, and its area and medium grey level on the negative image were measured. The enamel organ area demarcated by the microscopic field was also measured in order to establish the percentage of COX2 reactive area in each area analyzed. Finally, an overall parameter was established to integrate the two factors, by multiplying the grey value by the percentage of immunolabeled area^18,19^. We analyzed five sections for each incisor (20 sections per mouse, 120 sections for each study group).

### Statistical analysis

Results were expressed mainly as means +/− standard error of the mean (S.E.M.). The Kolmogorov-Smirnov normality test and the Levene variance homogeneity test were applied to the analysis of the element content in dental tissue. As the data did not show normal distribution and homogeneity of variance, significant differences were evaluated using the Kruskal-Wallis test (p < 0.05). All pair-wise multiple comparisons were performed using Dunn’s method.

## Results

### SEM-EDX analysis

The elements quantified were Ca, P, Al, K, Na, Mg and Cl. Groups treated with antibiotics did not show any significant differences in the content of the elements. Acetaminophen and celecoxib showed a significant decrease in Ca and P compared with the control samples. However, the decreases were proportional, and therefore the Ca/P ratios showed no significant differences (Table 1).

**Table 1:** Ca/P ratio and content of elements (expressed as % /w) present in murine molar (M3) enamel treated for 30 days with antibiotics and NSAIDs.

Treatment	Ca	P	Al	K	Na	Mg	Cl	Ca/P ratio
Control	46.94 ± 4.80	21.83 ± 2.54	0.53 ± 0.29	1.04 ± 0.36	0.62 ± 0.13	0.31 ± 0.06	0.31 ± 0.05	1.66 ± 0.09
Amoxicillin	43.06 ± 5.19	20.05 ± 1.50	0.41 ± 0.35	1.78 ± 1.47	0.62 ± 0.14	0.33 ± 0.11	0.30 ± 0.08	1.66 ± 0.20
Amox + Clavul	47.18 ± 6.14	22.37 ± 2.19	0.71 ± 0.37	1.16 ± 0.46	0.72 ± 0.26	0.31 ± 0.09	0.36 ± 0.08	1.63 ± 0.09
Erythromycin	44.79 ± 4.34	21.21 ± 2.07	0.67 ± 0.42	1.15 ± 0.40	0.62 ± 0.11	0.35 ± 0.08	0.34 ± 0.04	1.63 ± 0.09
Ibuprofen	42.00 ± 6.03	19.06 ± 1.31	0.39 ± 0.22	1.13 ± 0.51	0.50 ± 0.16	0.30 ± 0.24	0.34 ± 0.03	1.66 ± 0.18
Acetaminophen	36.73 ± 5.75^a^	17.92 ± 3.81^a^	0.32 ± 0.05	1.25 ± 0.45	0.53 ± 0.23	0.34 ± 0.14	0.30 ± 0.07	1.62 ± 0.35
Celecoxib	37.45 ± 6.61^a^	18.03 ± 2.82^a^	0.47 ± 0.12	1.16 ± 0.65	0.53 ± 0.18	0.29 ± 0.07	0.31 ± 0.05	1.60 ± 0.14

Ca: calcium; P: phosphorous; Al: aluminum; K: potassium; Na: sodium; Mg: magnesium; Cl: chlorine. ^a^p < 0.05 versus control.

### Immunohistochemistry

Mice in the groups treated with antibiotics and acetaminophen showed significantly lower amounts of immunoreactive COX2 at the enamel organ maturation stage of mouse incisors in comparison with the control and celecoxib groups. However, the ibuprofen- and celecoxib-treated groups showed no significant differences in immunoreactive COX2 content compared with the control teeth (Table 2).

**Table 2:** COX2 immunocytochemical quantification of the study groups.

Group	COX2 (grey value x % immunolabeled area)*
Control	41.07 ± 9.78
Amoxicillin	13.00 ± 1.89^a.b^
Amoxicillin/Clavulanate	13.27 ± 1.22^a.b^
Erythromycin	21.47 ± 3.65^a.b^
Ibuprofen	31.55 ± 5.66
Acetaminophen	14.11 ± 3.13^a.b^
Celecoxib	38.32 ± 4.55

^*^Negative images were used for immunocytochemical quantification (see material and methods section). Values expressed as mean +/− SEM of six animals. Differences: p < 0.05: ^a^COX2 content was lower in comparison with control samples: ^b^COX2 content was lower in comparison with celecoxib.

**Figure 1:**
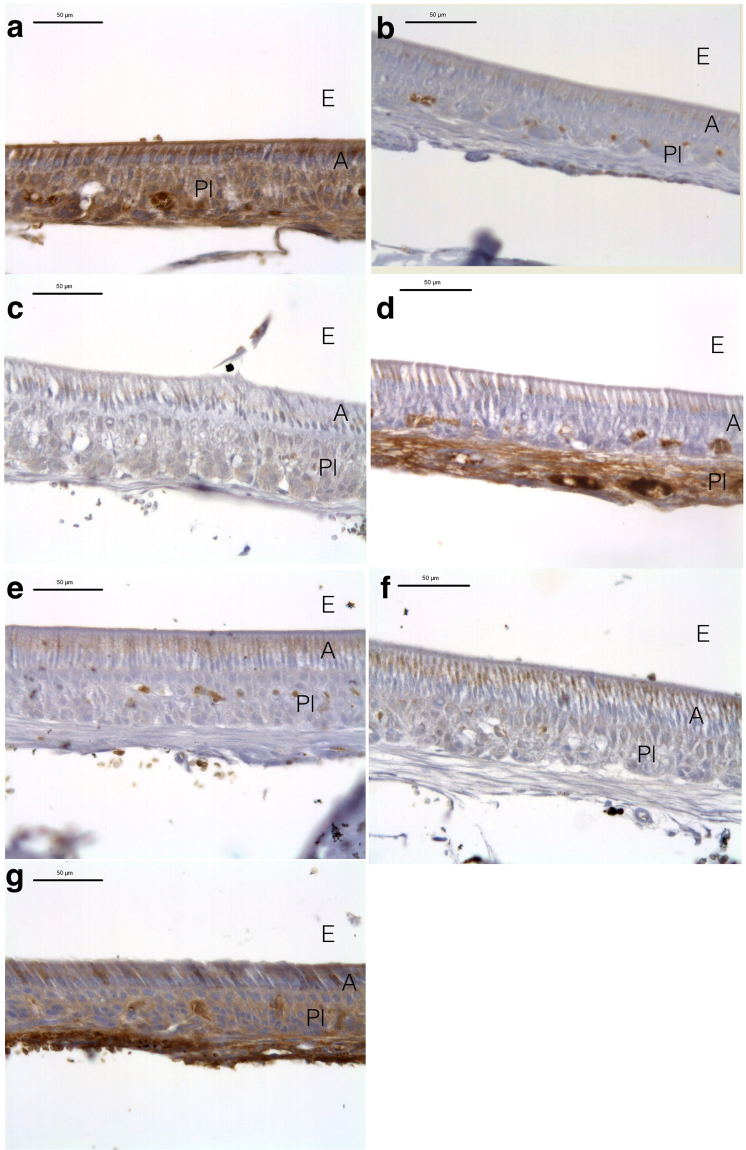
COX2 Immunostaining at the enamel organ maturation stage of mouse incisors. Intense immunoreactivity for COX2 is seen in the control (**a**) and celecoxib (**g**) groups. Weak immunoreactivity is shown in the amoxicillin (**b**), amoxicillin/clavulanate (**c**), erythromycin (**d**), and acetaminophen (**f**) groups and medium immunoreactivity in the ibuprofen (**e**) group. Note that COX2 immunoreactivity is indicated in three regions (ameloblast, papillary layer, and capillaries). A high degree of staining can be seen in the supranuclear region and apical cytoplasm of ameloblasts, while the basal region remains unlabeled. High reactivity is also seen inside the capillaries of the papillary layer, but diffuse staining is observed in the papillary layer proper. A: ameloblast, Pl: papillary layer, E: space occupied by enamel. Scale bar 50 μm.

Figure 1 shows immunostaining for COX2 in mouse incisor maturation-stage enamel. Intense COX2 immunoreactivity was detected in the control and celecoxib groups. Weak immunoreactivity was found in the amoxicillin, amoxicillin/clavulanate, erythromycin, and acetaminophen groups. Medium immunoreactivity was found in the ibuprofen group.

## Discussion

In mice, we studied the drugs most frequently administered to children during the first three years of life: the antibiotics amoxicillin, amoxicillin plus clavulanate, and erythromycin and the NSAIDs acetaminophen and ibuprofen^12,13^. We also studied celecoxib, a drug not prescribed in children, in order to inhibit COX2^15,16^.

Treatment was administered for 30-days, from days 21–51 postpartum. Most enamel mineralization takes place around this time in the murine third molar, and so this is the most convenient period to explore the possible effects of the different agents on the late phase of the maturation stage of amelogenesis^20^ and post-eruptive maturation^21^. During these 30 days, the total renovation of the mouse incisor occurs, making it possible to observe any possible alterations during the different stages of amelogenesis^22^.

The elements quantified in the EDX analysis were Ca, P, Al, K, Na, Mg and Cl. Ca and P are the most important elements as they comprise the majority of the tooth enamel’s mineral composition, forming the hydroxyapatite crystal network with the incorporation of small amounts of other elements^23^. The Ca and P content decreased with the administration of acetaminophen and celecoxib (Table 1). However, amoxicillin, amoxicillin plus clavulanate, erythromycin and ibuprofen produced no significant decrease in Ca and P levels, supporting the specific effect of acetaminophen and celecoxib. Nevertheless, the Ca/P molar ratio was maintained across all treatments in comparison with the control group. This indicates that even treatments that decreased the normal Ca and P content did not alter the standard Ca/P ratio, which is approximately 1.67 [Ca_10_(PO_4_)_6_(OH)_2_]^24^.

NSAIDs are known to inhibit cyclooxygenases^25^, enzymes that catalyze the formation of prostaglandins from arachidonic acid by mediating several physiological processes, including inflammation. There are two COX isozymes: COX1 is the constitutive enzyme, whereas COX2 is the inducible isozyme, whose expression is transiently increased in response to a number of pro-inflammatory stimuli. COX2 induction results in a rapid augmentation of intracellular nitric oxide levels, cytokines, and Ca^2+^ intracellular influx^26^. It is reasonable to hypothesize that enamel mineralization would require mediators of inflammation due to the high ion influx requirement of ameloblasts during crystal formation. Therefore, we explored the involvement of COX2 in enamel maturation in mouse incisors and its correlation with possible changes in Ca and P content after administration of the study drugs.

Our results showed that COX2 was present in the enamel organ. As far as we are aware, this is the first time the presence of COX2 in enamel has been detected. However, the quantification of immunoreactive COX2 followed a complex pattern. After 30 days chronic treatment with antibiotics, the content of COX2 in the enamel organ decreased (Table 2). Acetaminophen also provoked a significant decrease in immunoreactive COX2. Ibuprofen produced a small, non-significant decrease (31.55 ± 5.66 vs. 41.07 ± 9.78 in control mice). Celecoxib did not alter the amount of immunoreactive COX2 in comparison with control samples (Table 2, Fig. 1); given that treatment with both acetaminophen and celecoxib decreased Ca and P content in enamel, this finding for celecoxib – a selective COX2 inhibitor^15,16,25^ – is, at first glance perplexing. However, Vardar-Sengul *et al*.^27^ reported that celecoxib inhibits COX2 activity but not its expression. Therefore it may be inferred that, even though the amount of enzyme stained by a COX2-specific antibody in immunochemical preparations was similar to that of the untreated control group, COX2 activity would be lower during celecoxib administration. This would account for the involvement of COX2 in normal enamel mineralization, even though it represents a limitation to the use of celecoxib as a control of COX2 inhibition in histochemical studies where the amount of protein rather than activity is determined. Inhibition of COX2 activity by celecoxib would produce a reduction in the prostacyclin, PGI2, which would decrease blood flow in the ameloblast area. The rapid diffusion of nutrients into the ameloblast layer is necessary during the enamel maturation stage, as is the rapid incorporation of ions required for correct crystal growth^28^. A reduction in PGI2 might also alter the fluid-buffering capacity of local tissue involved in the maintenance of pH homeostasis in the mineralizing enamel matrix environment, which is essential for building normal enamel^29^. COX1 and COX2 are enzymes with topologically-similar active sites^25,30^ and therefore totally selective inhibition of COX1 and COX2 is not possible with NSAIDs usually prescribed in children. However, recent research indicates that acetaminophen acts as a preferential COX2 inhibitor; in humans, acetaminophen (1000 mg, single dose) produces 83% COX2 inhibition but only 56% inhibition of COX1^26,31^ but ibuprofen (800–1,200 mg/day) showed a lower potency of COX2 inhibition and a similar effect on constitutive COX1 and inducible COX2^27^. In this context, our results indicate a clear correlation between COX2 inhibition and reductions in Ca and P. Acetaminophen had a significant effect on both parameters (Tables 1 and 2), but ibuprofen showed a non-significant reduction in COX2 and no effect on the Ca and P content.

The effects of the antibiotics tested were complex, as the three treatments tested reduced immunoreactive COX2 (Table 2) but did not provoke any significant effect on the normal Ca and P content of enamel (Table 1). Erythromycin is a macrolide that inhibits protein synthesis dose-dependently^32^. It is more effective in bacterial and mitochondrial protein synthesis, but chronic administration could also affect the synthesis of inducible enzymes with a relatively short half-life, such as COX2. It is reported that its use during the first years of life increases MIH in children. A significantly-higher risk of enamel defects was also noted in the first permanent molars of children with higher intakes of macrolide antibiotics during the first years of life^10,33^. There are no previous studies of the effects of erythromycin on enamel in animal models, but our results suggest that the hypomineralization provoked by erythromycin could be related to enamel thickness rather than the Ca and P content or the Ca/P ratio.

Amoxicillin, whether alone or supplemented with clavulanate, did not produce any effects. The decrease observed in immunoreactive COX2 is difficult to explain in terms of the inhibition of protein synthesis, as neither amoxicillin nor clavulanate act on this process. In any case, the reduction observed in COX2 did not correlate with significant decreases in Ca and P in murine incisor samples. However, several studies have described the effect of amoxicillin on enamel maturation. It is reported that chronic administration of amoxicillin/clavulanate in mice affects ameloblast functions during the maturation phase, causing detachment from the enamel matrix, hypomineralization and reductions in P and Ca^4^; the severity of the effect is dose-dependent. Gottberg *et al*.^34^ reported hypomineralization in all rats in a group treated with 100 mg/kg amoxicillin, while 50 mg/kg only caused hypomineralization in 50% of the animals. Even a single, very-high dose (3 g/Kg/day) of amoxicillin affects normal tooth dentin mineralization specifically, but does not affect enamel mineralization in rat incisor odontogenesis^35^. Irrespective of the high doses needed to provoke hypomineralization, our results indicate that no effects were observed in terms of Ca/P ratio or Ca and P content. However, recent studies have reported some effects of amoxicillin on other parameters, such as a decrease in the thickness of the enamel matrix^36^. Nevertheless, this effect of amoxicillin on enamel thickness is not clear, as Laisi *et al*.^10^ reported that the antibiotic increased enamel thickness in children.

In conclusion, our results suggest that COX2 is involved in the normal maturation stage of the enamel organ and the control of calcium and phosphorus levels in the mature enamel. The consumption of acetaminophen (an inhibitor of COX2 activity) during the formation of the enamel could result in hypomineralization. Amoxicillin, amoxicillin/clavulanate and erythromycin administration reduces the quantity of enzyme present in the enamel organ during the maturation period, but not its activity.

Experimental studies of the combination of these drugs in healthy animals and animals with infections or fever, etc., are required to determine whether they produce effects that, summed together, may cause MIH.
